# Determination of the differential expression of mitochondrial long non-coding RNAs as a noninvasive diagnosis of bladder cancer

**DOI:** 10.1186/1471-2490-12-37

**Published:** 2012-12-18

**Authors:** Alexis Rivas, Verónica Burzio, Eduardo Landerer, Vincenzo Borgna, Sebastian Gatica, Rodolfo Ávila, Constanza López, Claudio Villota, Rodrigo de la Fuente, Javiera Echenique, Luis O Burzio, Jaime Villegas

**Affiliations:** 1 Andes Biotechnologies S.A. and Fundación Ciencia para la Vida, 7780272, Santiago, Chile; 2 Departamento de Ciencia Biológicas, Facultad de Ciencias Biológicas, Universidad Andrés Bello, 8370146, Santiago, Chile; 3 Facultad de Medicina, Universidad Andrés Bello, 8370146, Santiago, Chile; 4 Urology Unit, Clínica Indisa, 7520440, Santiago, Chile; 5 Urology Unit, Hospital Barros Luco Trudeau, 8900085, Santiago, Chile

## Abstract

**Background:**

Bladder cancer is a significant cause of morbidity and mortality with a high recurrence rate. Early detection of bladder cancer is essential in order to remove the tumor, to preserve the organ and to avoid metastasis. The aim of this study was to analyze the differential expression of mitochondrial non-coding RNAs (sense and antisense) in cells isolated from voided urine of patients with bladder cancer as a noninvasive diagnostic assay.

**Methods:**

The differential expression of the sense (SncmtRNA) and the antisense (ASncmtRNAs) transcripts in cells isolated from voided urine was determined by fluorescent *in situ* hybridization. The test uses a multiprobe mixture labeled with different fluorophores and takes about 1 hour to complete. We examined the expression of these transcripts in cells isolated from urine of 24 patients with bladder cancer and from 15 healthy donors.

**Results:**

This study indicates that the SncmtRNA and the ASncmtRNAs are stable in cells present in urine. The test reveals that the expression pattern of the mitochondrial transcripts can discriminate between normal and tumor cells. The analysis of 24 urine samples from patients with bladder cancer revealed expression of the SncmtRNA and down-regulation of the ASncmtRNAs. Exfoliated cells recovered from the urine of healthy donors do not express these mitochondrial transcripts. This is the first report showing that the differential expression of these mitochondrial transcripts can detect tumor cells in the urine of patients with low and high grade bladder cancer.

**Conclusion:**

This pilot study indicates that fluorescent *in situ* hybridization of cells from urine of patients with different grades of bladder cancer confirmed the tumor origin of these cells. Samples from the 24 patients with bladder cancer contain cells that express the SncmtRNA and down-regulate the ASncmtRNAs. In contrast, the hybridization of the few exfoliated cells recovered from healthy donors revealed no expression of these mitochondrial transcripts. This assay can be explored as a non-invasive diagnostic tool for bladder cancer.

## Background

Bladder cancer (BC) is an important cause of morbidity and mortality, with an estimated 386.000 new cases and 150.000 deaths occurring worldwide in 2008 [1]. Bladder tumors are classified into four categories: papilloma, papillary urothelial carcinoma of low malignant potential, low-grade carcinoma, high-grade carcinoma and carcinoma *in situ*[2]. About 90% of bladder cancers are urothelial carcinomas and transitional cell carcinomas (TCC) and the rest include squamous cell carcinomas and adenocarcinomas. As many other types of cancer, early detection of BC will allow effective treatments of patients, improving long-term survival.

The “gold standard” in the detection of BC is cystoscopy. This examination, however, is unpleasant, time consuming, expensive and may result in infections and urethral damage [3]. On the other hand, urine cytology has high specificity but low sensitivity, especially in low-grade disease [4,5]. To improve the detection of BC cells in voided urine, several tumor markers and tests have been developed [6,7]. One of these tests is based on fluorescent *in situ* hybridization (FISH) to detect chromosomal alterations characteristic of BC [8].

Human cells express a family of mitochondrial long non-coding RNAs (ncRNA) containing stem-loop structures. One of these transcripts, the sense mitochondrial ncRNA or SncmtRNA, is expressed in normal proliferating cells and tumor cells but not in non-dividing cells [9,10]. Experimental evidences suggest that this transcript plays a regulatory role of the cell cycle [11]. In addition, normal human proliferating cells in culture or in normal human tissues express two antisense transcripts, AsncmtRNA-1 and AsncmtRNA-2 [10]. Interestingly, the SncmtRNA and the AsncmtRNAs exit the mitochondria and localize to the cytoplasm and the nucleus in association with chromatin and nucleoli, suggesting that the function of these transcripts take place outside the organelle [12].

The function of the ASncmtRNAs is less clear. However, an interesting observation is that the ASncmtRNAs are down-regulated in tumor cell lines as well as in tumor cells present in different types of human cancer and patients [10]. *In situ* hybridization of twelve BC biopsies from different patients shows expression of the SncmtRNA and down-regulation of the ASncmtRNAs [10]. Since down-regulation of the ASncmtRNAs seems to be independent of the tissue of origin of tumor cells, the differential expression of these transcripts can be applied as a cancer diagnostic method for cells in suspension. Here, we present a one-tube fluorescence *in situ* hybridization protocol applied to cells in suspension (S-FISH), that takes about 60 min to perform and using simultaneously labeled probes for both SncmtRNA and AsncmtRNAs. This method was applied to cells isolated from urine of patients with bladder cancer (BC). In twenty four patients with low and high grade of BC, S-FISH revealed cells expressing the SncmtRNAs and not the ASncmtRNAs, hence corresponding to cancer cells phenotype. The expression of these transcripts was negative in the few cells isolated from the urine of healthy donors. The differential expression of the SncmtRNA and the ASncmtRNAs in cells isolated from voided urine can be explored as a new non-invasive diagnostic test for BC.

## Methods

### Tumor cell culture

T24 and RT4 cells (human bladder carcinoma) and DU-145 cells (prostate carcinoma) were cultured according to ATCC recommendations. Cultures were maintained in a humidified incubator at 37°C and 5% CO_2_. Peripheral blood mononuclear cells (PBMC) from healthy donors were isolated and stimulated with phytohaemagglutinin (PHA) for 48 h as described before [9,10,13]. Primary renal mixed epithelial cells were obtained from ATCC and cultured according to ATCC guidelines.

### S-FISH

All the hybridization steps were performed in MaxiRecovery™ tubes of 0.5 ml (Axygen Scientific, US). After trypsinization (Invitrogen, Carlsbad, US), about 10^5^ cells were recovered by centrifugation at 200 × *g* for 10 min at room temperature (RT). The cell pellet was resuspended in 100 μl HCl 0,2 N and incubated for 5 min at RT. Afterwards, the cell suspension was diluted with 400 μl PBS (50 mM sodium phosphate, 150 mM NaCl and 2 mM EDTA, pH 9.0) and centrifuged again. The sediment was resuspended in 100 μl hybridization buffer (50% formamide, 150 μg/ml herring sperm DNA, 4X SSC, 2 mM EDTA) containing 0,5 μM 5′-Alexa fluor 488-labeled probe P1 (5′ GTTCTTGGGTGGGTGTGGG 3′), complementary to the SncmtRNA and 0,05 μM each of two 5′ Texas Red-labeled probes P2 (5′ GATAACAGCGCAATCCTATT 3′) and P3 (5′ ACCGTGCAAAGGTAGCATAATCA 3′), complementary to the ASncmtRNAs. In addition, two negative controls corresponding to mismatch probes P5 for the SncmtRNA (MM: 5′ TTTATTTGATGAGTGTGAG 3′), labeled with Alexa fluor 488 and probe P6 for the ASncmtRNAs (MM: 5′ GTAAAGATAGTATAATAATTTATTAATTAAATATA 3′), labeled with Texas Red at the 5′ end. The labeled probes were obtained from Invitrogen (Carlsbad, CA, USA).

Hybridization was carried out for 15 min to 2 h at 37°C. The final wash was performed by addition of four volumes of stringency buffer (2X SCC + 2 mM EDTA) to the hybridization mix, incubated for 5 min at 37°C and finally centrifuged at 200 × *g* for 10 min. The supernatant was discarded and a small volume of approximately 20 μl of the residual supernatant was left in the tube to resuspend the cells. The cells were finally stained in a solution of 1 μg/ml DAPI, deposited onto a positively charged slide (Thermo Scientific, US) and mounted in fluorescent medium (DAKO). Samples were analyzed by fluorescence microscopy on an Olympus BX-51 microscope under x600 magnification, with 300-600 ms exposition and results were documented with Q-capture Pro software. The positive hybridization control corresponded to a 5′-Texas Red-labeled probe complementary to 18S rRNA (P4: 5′ AGTGGACTCATTCCAATTACA 3′).

### Voided urine

About 30-50 ml voided urine from male and female healthy donors was carried out in agreement with the ethical guidelines approved by the Ethical Committee of the Fundacion Ciencia para la Vida. The urine from healthy donors (50 ml) was loaded with 5×10^4^ to 1×10^5^ T24 or DU-145 cells or PHA-stimulated lymphocytes and incubated at 4°C for 24 h. The cells were sedimented by centrifugation at 700 × *g* for 10 min. The supernatant was discarded, leaving only 5 ml, which were transferred to a Kova tube (Hycor Biomedical Inc., US) and centrifuged again at 200 × *g* for 10 min at RT. Most of the supernatant was discarded and the remnant (~1ml) was transferred to a 0,5 ml Maxy-Recovery tube (Axygen Scientific, US), centrifuged at 200 × g and subjected to S-FISH as described above.

Twenty four patients diagnosed with BC were recruited at the Urology Unit of the Hospital Barros Luco Trudeau and Clinica Indisa (Santiago, Chile). The urine samples were obtained with informed consent under the Ethical Regulations of the Hospital Barros Luco Trudeau and Clinica Indisa and with the approval of the Ethic Committee of Fundacion Ciencia para la Vida. The tumor biopsies were graded as reported [14] and the data are summarized in Table 1. The first-morning voided urine (50 ml) was collected and stored at 4°C in a cooler and transported to the laboratory. The urine was centrifuged at 700 × *g* for 10 min within 4 h after collection and S-FISH was performed as described above. Parallel samples were stained with hematoxylin.

**Table 1:** Distribution of tumor stage and grade among all patients included in this study

**Grade and stage**					
**distribution**	**None**	**Grade 1**	**Grade 2**	**Grade 3**	**Total**
No tumor	15				15
Ta		11	2	4	17
T1		1	1	1	3
T2-4				3	3
CIS				1	1
**Total**	**15**	**12**	**3**	**9**	**39**

## Results

### Optimization of the S-FISH protocol

**Figure 1:**
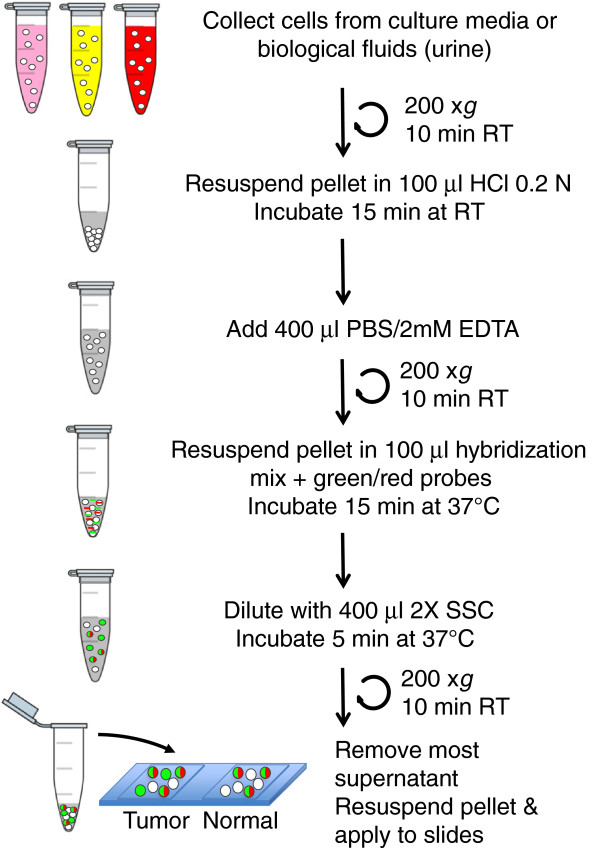
**Schematic representation of S-FISH of cells from cultures or present in biological fluids.** Cells are collected by centrifugation at 200 x *g* for 10 min and the pellet suspended in 100 μl 0.2 N HCl. After washing with PBS, the cells are hybridized in 100 μl hybridization solution containing fluorescent-labeled probes for the SncmtRNA and the ASncmtRNAs. After a stringency washing, the cells are analyzed by fluorescence microscopy.

**Figure 2:**
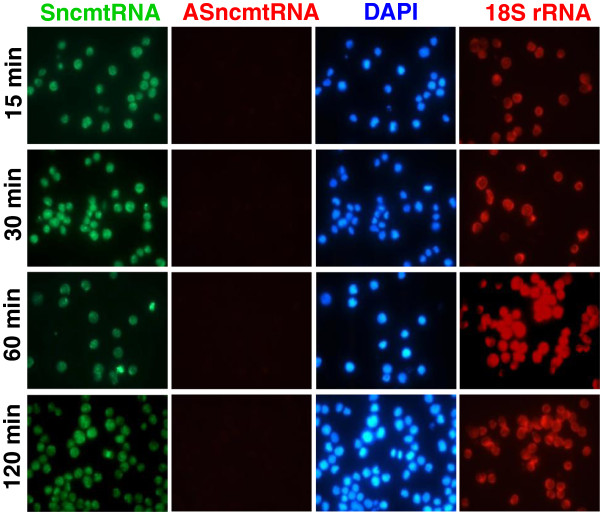
**Hybridization kinetics on T-24 cells.** After trypsinization, 10^5^ cells were collected by centrifugation at 200 x *g* for 10 min and the pellet was subjected to S-FISH as indicated in Figure 1. Cells were hybridized simultaneously with probes complementary to the SncmtRNA and ASncmtRNAs for 15, 30, 60 and 120 min. Only the green signal corresponding to the expression of the SncmtRNA was observed, while expression of the ASncmtRNAs was down-regulated (red fluorescence). Cell distribution was revealed with DAPI staining (DAPI). The positive control corresponding to Texas red-labeled 18S rRNA-probe was run in parallel (60x).

A schematic representation of the S-FISH is shown in Figure 1. Briefly, cells were collected by centrifugation from cell culture or from biological fluids such as urine and blood, followed by permeabilization with 0.2 N HCl. After neutralization, the cells were recovered, hybridized with a set of probes labeled with fluorophores, washed and analyzed by fluorescence microscopy (see Methods). To determine the minimum hybridization time needed for the detection of the mitochondrial ncRNAs, T-24 cells (bladder carcinoma cell line) were subjected to S-FISH for different time periods. After 15 minutes of hybridization the fluorescent signal of the SncmtRNA was as strong and specific as longer hybridization times (Figure 2, SncmtRNA). The red signal corresponding to the ASncmtRNA was negative at any of the hybridization times tested confirming the tumor pattern of expression of T-24 cells (Figure 2, ASncmtRNAs). The 18S rRNA used as positive control was expressed in all cells and the corresponding red signal was also independent of hybridization times ranging from 15 to 120 min (Figure 2, 18S rRNA, red). The same results were obtained with the bladder carcinoma cell line RT4 (unpublished data).

### Stability of the mitochondrial ncRNAs in urine

**Figure 3:**
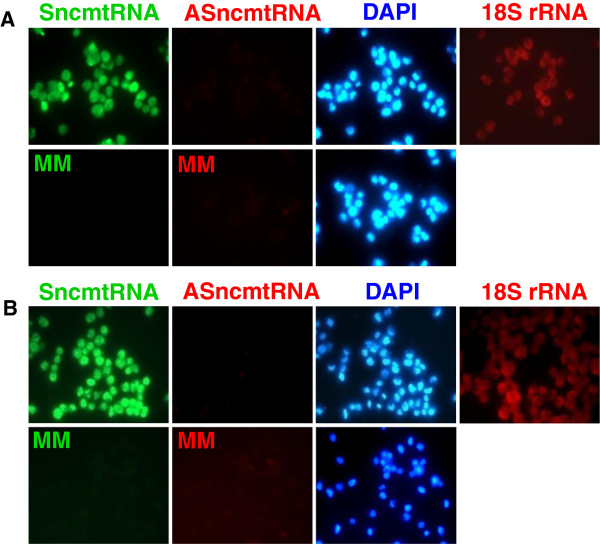
**Stability of the mitochondrial ncRNAs in urine.** A, 5 × 10^4^ T-24 cells were loaded in urine obtained from healthy donors and stored at 4°C for 24 h. Cells were then recovered by centrifugation as described and subjected to S-FISH. Only the SncmtRNA (green fluorescence) was detectable, while probes for ASncmtRNAs (red fluorescence) showed absence of signal, as did the corresponding mismatch probes (MM, green or red). The cells were also counterstained with DAPI. A parallel sample was hybridized with a probe specific for the 18S rRNA (18S rRNA). B, DU-145 cells (prostate carcinoma) were subjected to the same S-FISH protocol. Notice that the cells only express the SncmtRNA. No fluorescent signal was obtained with the probes targeted to the ASncmtRNA or with MM probes.

An interesting model to test S-FISH was cancer cells obtained from voided urine of patients with BC. Since the stability of cells and their RNAs in urine is uncertain, we asked whether the urine would affect the stability of the SncmtRNA and the ASncmtRNAs from tumor cells and normal proliferating cells. Fresh urine of healthy donors was loaded with 5×10^4^ T-24 cells/ml and maintained at 4°C for 24 h. Cells were then recovered by centrifugation and subjected to S-FISH as described. Analysis of several fields indicated that all cells were positive for the SncmtRNA and the intensity of the fluorescent signal was comparable to that of fresh T-24 cells (compare Figures 2 and 3, SncmtRNA). The red hybridization signal corresponding to the ASncmtRNAs (Figure 3A, ASncmtRNAs) was negative and comparable to the negative green fluorescence of the mismatch (MM) probes for the sense transcript (Figure 3A, panels MM green) or the red fluorescence of the MM probe for the antisense transcripts (Figure 3A, panels MM red). The hybridization signal of 18S rRNA was similar to that of fresh T-24 cells (Figure 3A, 18S rRNA). The same results were obtained with DU-145 cells (prostate carcinoma cell line) (Figure 3B).

**Figure 4:**
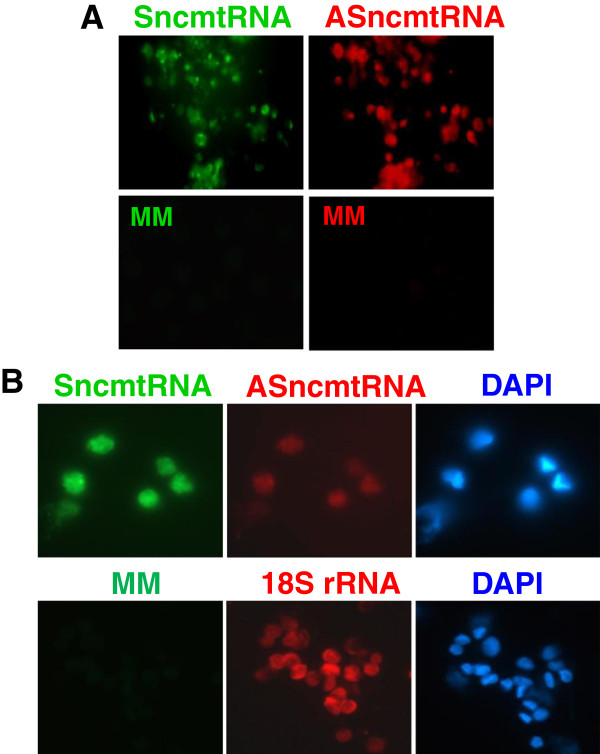
**Normal proliferating cells express the SncmtRNA and the ASncmtRNAs.** A, PHA-stimulated human lymphocytes were hybridized simultaneously with probes complementary to the SncmtRNA and ASncmtRNA. After 15 min of hybridization, strong signals were obtained with both probes in the same cells. No signal was observed with the corresponding mismatch probes (MM, green or red fluorescence). (60x). B, Kidney epithelial cells express both mitochondrial transcripts. The S-FISH revealed that the same cells (as revealed by DAPI staining) expressing the SncmtRNA also express the ASncmtRNAs. The mismatch control (MM) was negative and 18S probe was used as a positive control (60x).

In addition, we determined the stability of the SncmtRNA and the ASncmtRNAs in normal proliferating cells maintained in urine for 24 h at 4°C. Isolated PBMC were activated with PHA and incubated in urine for 24 h previous to S-FISH. PHA-stimulated PBMCs showed a positive signal for both the SncmtRNA and the ASncmtRNAs (Figure 4A) confirming the expression pattern of normal proliferating cells [10]. On the other hand, the hybridization signals were negative with the MM probe to either the SncmtRNA (green fluorescence) and the ASncmtRNAs (red fluorescence) (Figure 3A, MM). Similarly, the same normal renal epithelial cells (DAPI staining) expressing the SncmtRNA were also expressing the ASncmtRNAs (Figure 4B). The green mismatch control was negative (Figure 4B).

### Detection of tumor cells in voided urine of patients with BC

**Figure 5:**
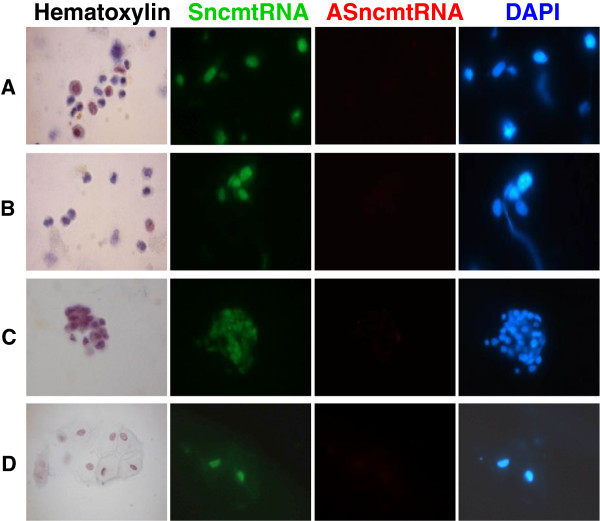
**Representative S-FISH of tumor cells obtained from urine from patients with BC.** Cells were collected by centrifugation from three grade 3 BC patients (A to C) and one grade 2 BC patient (D). The cells were hybridized simultaneously with probes complementary to the SncmtRNA and the AsncmtRNAs. After hybridization, cells were stained with DAPI. Only the SncmtRNA was detected in cells from the four samples. A parallel sample was stained with H&E (60×).

Then we asked whether S-FISH can be applied to tumor cells present in voided urine obtained from 24 patients with BC diagnosed by cystoscopy and confirmed by biopsy. Table 1 show the grade and stage distribution of the samples. The cells were recovered from urine 4 h after collection and subjected to S-FISH as described before. Then, 25 fields of each sample at 40x magnification were analyzed and recorded. In all 24 urine samples, the S-FISH detects cells expressing the SncmtRNA and down-regulate the ASncmtRNAs. As described before, this expression pattern corresponds to a cancer cells. Figure 5 illustrates S-FISH results obtained with urine cells recovered from four patients with BC. Samples A, B and C correspond to urine cells recovered from patients with grade 3 BC (Figure 5). Samples D corresponds to cells obtained from patients with grade 2 (Figure 5). It is important to mention that the cellularity of samples C and D was low. Although the cellularity of grade 1 BC was low, the S-FISH detected few cells that only express the SncmtRNA. In six urine samples obtained from the 15 healthy donors (Table 1) few cells were recovered. However, the hybridization signals indicate that the SncmtRNA and the ASncmtRNAs were down-regulated. The typical debris present in some urine samples did not interfere with the hybridization signal.

## Discussion

FISH provides an important tool for conventional cytogenetics and evaluation of chromosomal abnormalities associated with several malignancies [15]. Some examples of chromosomal abnormalities are found in several diseases, such as BC [16-20], multiple myeloma [21-23], breast cancer [24], hematological malignancies [25-28] and lung cancer [29,30] among others. Different types of tumor require specific sets of probes corresponding to particular chromosomal deletions/translocations characteristic of each cancer. Sokolova *et al.* reported the development of a FISH assay with high sensitivity and specificity for high grade BC using four labeled probes specific for the pericentromeric regions of chromosomes 3, 7 and 17 and for the detection of the 9p21 deletion [8]. These results were confirmed in later studies with a large cohort of BC patients [16-20] using several probes combined into a single multiprobe cocktail, to detect polysomy of chromosomes 3, 7 and 17 and homozygous deletion of 9p21 in the urine of BC patients (Urovysion, Abbot Molecular/Vysis, Des Plaines, IL). However, this test has low sensitivity for low-stage and low-grade tumors, which are the main group that recur [3].

The S-FISH assay described here is able to detect the differential expression of the SncmtRNA and the ASncmtRNAs in normal and cancer cells. This a simple protocol that was optimized in three steps including a single permeabilization step with HCl, a short hybridization step and a brief washing that basically involves the dilution of the hybridization mix with stringency buffer (Figure 1). The protocol contains only three centrifugation steps in the same tube, minimizing the manipulation of cells and therefore maximizing RNA preservation and cell recovery. This test is reproducible and has been applied to other normal and tumor cell lines. Hybridization of normal proliferating cells (human umbilical vein endothelial cells, keratinocytes and melanocytes) reveals the expression of the SncmtRNA and the ASncmtRNAs. In other human tumor cell lines such as HeLa, 42/95 and SK-MEL-2 (melanoma), Jurkat and HL-60 (leukemia) and MDA-MB-231 (breast carcinoma), S-FISH revealed expression of the SncmtRNA and down-regulation of the ASncmtRNA (unpublished data).

Moreover, S-FISH was able to detect cancer cells in urine from twenty four patients with BC and the results were independent of the grade of BC and the urine cellularity (see Figure 5). Taken together, the results suggest that the diagnostic test has a very high positive outcome independent of the grade and the amount of cells recovered from urine of patients with BC. In the urine from healthy donors, cells were recovered only from six out of fifteen samples and the S-FISH revealed absence of signal to both transcripts.

## Conclusions

Taken together, this pilot study suggests that S-FISH could be used for detection and regular surveillance programs of patients with BC. Interestingly, the results indicate that the exfoliated bladder tumor cells from low and high grade BC conserve the expression pattern observed in bladder cancer biopsies: expression of the SncmtRNA and down-regulation of the ASncmtRNAs. In summary, S-FISH may potentially be used as a non-invasive diagnostic test for bladder cancer. However, to validate the test, a large cohort of patients with low-grade and high-grade neoplasms should be included together with other urological diseases such as glomerulonephritis, infections of the upper urinary track and other benign urinary track diseases.

## Competing interests

Authors report no competing interests

## Authors’ contributions

LOB, VB and JV conceived the experimental plan, analyzed the data, and drafted the manuscript. AR, VB, EL, VB, SG, RA, CL, CV, RF, and JE carried out the experiments. RF, EL and VB reviewed the patients’ history and pathological data. All authors read and approved the final manuscript.

## Pre-publication history

The pre-publication history for this paper can be accessed here:

http://www.biomedcentral.com/1471-2490/12/37/prepub
